# Case report: Area of focus in a case of malignant hypertension

**DOI:** 10.3389/fcvm.2022.1108666

**Published:** 2023-01-13

**Authors:** Francesca Gaia Bosisio, Desirè Mingardi, Elisabetta Moretti, Giorgia Muzi, Francesco Russomanno, Nicola Tassani, Deborah Stassaldi, Claudia Agabiti Rosei, Carolina De Ciuceis, Massimo Salvetti, Maria Lorenza Muiesan

**Affiliations:** ^1^UOC 2 Medicina, ASST Spedali Civili di Brescia, Brescia, Italy; ^2^Emergency Medicine ASST Spedali Civili Brescia, Brescia, Italy; ^3^Department of Clinical and Experimental Sciences, University of Brescia, Brescia, Italy

**Keywords:** malignant hypertension, hypertensive emergency, organ damage, atypical hemolytic uremic syndrome, thrombotic microangiopathies, left ventricular hypertrophy, case report

## Abstract

Malignant hypertension (MH) is characterized by severe hypertension (usually grade 3) associated with fundoscopic changes (flame hemorrhages and/or papilledema), microangiopathy and disseminated intravascular coagulation. In addition encephalopathy, acute heart failure and acute deterioration in renal function may be present. The term “malignant” reflects the very poor prognosis for this condition if untreated. When severe hypertension is associated with hypertension-mediated organ damage (HMOD) a life-threatening situation that requires immediate but careful intervention occurs (hypertensive emergency). In the last few years an increase in the number of patients with malignant hypertension has been observed, especially among those patients with black ethnicity. Limited access to treatment and the poor adherence to anti-hypertensive therapy may contribute to the development of hypertensive emergencies. It is considered appropriate to study patients in order to rule out thrombotic thrombocytopenic purpura and hemolytic uremic syndrome. In fact, the microvascular damage caused by malignant hypertension can favor intravascular hemolysis like Thrombotic Microangiopathies (TMs). TMs may present in three different clinical conditions: typical hemolytic uremic syndrome (HUS), atypical hemolytic uremic syndrome (aHUS) and thrombotic thrombocytopenic purpura (TTP). TMs can arise in the context of other pathological processes, including malignant hypertension.

## Introduction

**FIGURE 1:**
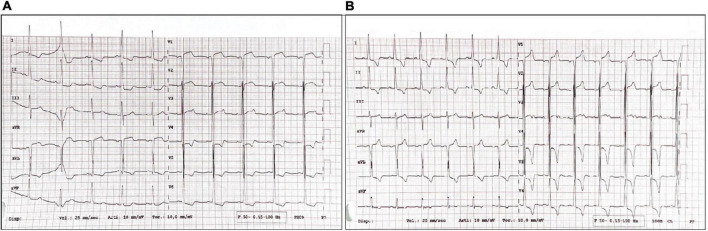
**(A)** ECG at Emergency Department (ED) admission. **(B)** ECG a few days after hospitalization.

A 46-years old man, smoker, without previous diseases in his past medical history, not taking any medications at the time of hospitalization and with a family history of diabetes and hypertension, was admitted to the Emergency Department (ED) of a spoke hospital. At admission he was complaining chest pain, headache and vomiting starting a few days before admission. At ED admission vital parameters were as follows: heart rate (HR) 81 bpm, blood pressure (BP) 176/101 mmHg, peripheral arterial oxygen saturation (SpO2) 99% with O2 therapy (3 L/min) and a Glasgow Coma Scale (GCS) value of 15 without other significant clinical or neurological findings. Laboratory investigations showed mild reduction of hemoglobin value (10 g/dL) with normal mean corpuscular volume (91.6 fL), thrombocytopenia (50,000/dL), severe increase of creatinine (6.52 g/dL), mild hypokalemia (2.9 mEq/L), normal sodium value (138 mEq/L), and severe increase of Troponin-T (215 ng/L). The ECG showed ST-elevation in precordial leads V1-V2, Sokolow–Lyon index 46 mm, left ventricular hypertrophy (LVH), and strain pattern (down sloping convex ST segment with an inverted asymmetrical T-wave opposite to the QRS axis) (Figure 1A). Chest X-ray showed cardiomegaly (Cardio-Thoracic index 0.61) and brain computerized tomography did not highlight any significant findings.

## Case description

**FIGURE 2:**
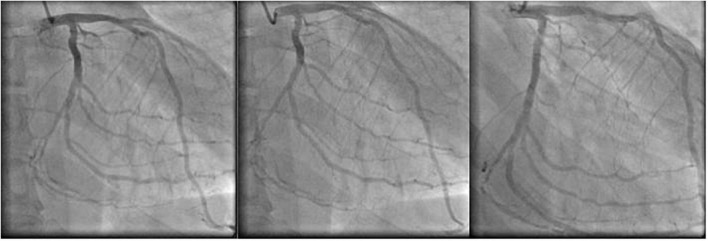
Coronary angiography: sequence of different frames.

Based on the clinical suspicion of acute coronary syndrome, the patient was transferred to the Intensive Care Unit (ICU) of the hub hospital ASST Spedali Civili in Brescia. A transthoracic echocardiogram confirmed increased thickness of left ventricular (LV) walls [interventricular septum (IVS) 21 mm, posterior wall (PW) 20 mm], “super” normal left ventricular ejection fraction (LVEF) 70%, wall motion abnormalities (basal and middle antero-lateral segments akinesia and posterior basal portion hypokinesia) and suspect of infiltrative pathology. Coronary angiography was performed, showing moderate coronary artery disease, i.e., 40% stenosis of the anterior interventricular artery, 50% stenosis of the third branch of the left marginal artery and coronary sclerosis of the posterolateral branch of the circumflex artery and of the third marginal branch of the right posterolateral artery (Figure 2).

Based on the coronary angiography results, other invasive treatment was excluded. Chest pain disappeared after nitrate i.v. administration.

In order to reduce BP, intravenous nitroprusside first and nitrate thereafter were given, with a sudden decrease of systolic and diastolic BP values (from 200/100 to 130/80 mmHg). Concomitantly negative hyperacute T waves in the anterior leads were observed at ECG, suggesting myocardial injury, worsened by the rapid fall in BP (Figure 1B).

The patient was transferred to the high intensity ward of the Internal Medicine Department for further diagnostic and therapeutic management.

Laboratory findings showed low platelet count (nadir 50,000/dL), anemia with high lactate dehydrogenase (LDH), reduced haptoglobin and schistocytes at the peripheral blood smear, all suggesting thrombotic microangiopathy (TMA), in the presence of severe hypertension and multiple organ damage (acute kidney injury and myocardial damage). Complement dysregulation, based on C5b-9 or membrane attack complex (MAC) of 475 ng/ml (normal values between 140 and 280 ng/ml) was detected. Plasma renin and aldosterone were measured in addition to urinary metanephrines and aldosterone and renin were elevated (aldosterone 44,5 ng/dL, renin 81,8 uUI/mL). Cortisol and thyroid stimulating hormone (TSH) were in physiological range.

A renal arteries ultrasound was performed in the suspicion of renovascular hypertension; no renal arteries stenosis was observed, although resistance indices were increased in the right interlobar arteries.

In order to rule out a possible auto-immune diseases, antinuclear antibodies (ANA), extractable nuclear antigen antibodies (ENA), anti-neutrophil cytoplasmic antibodies (ANCA), anti-dsDNA and anticardiolipin antibodies have been also dosed, and the Coombs test has been performed.

A brain magnetic resonance (MRI) ruled out posterior reversible encephalopathy syndrome (PRES), but showed the presence of small areas of aspecific gliosis in the white fronto-temporal lobes.

**FIGURE 3:**
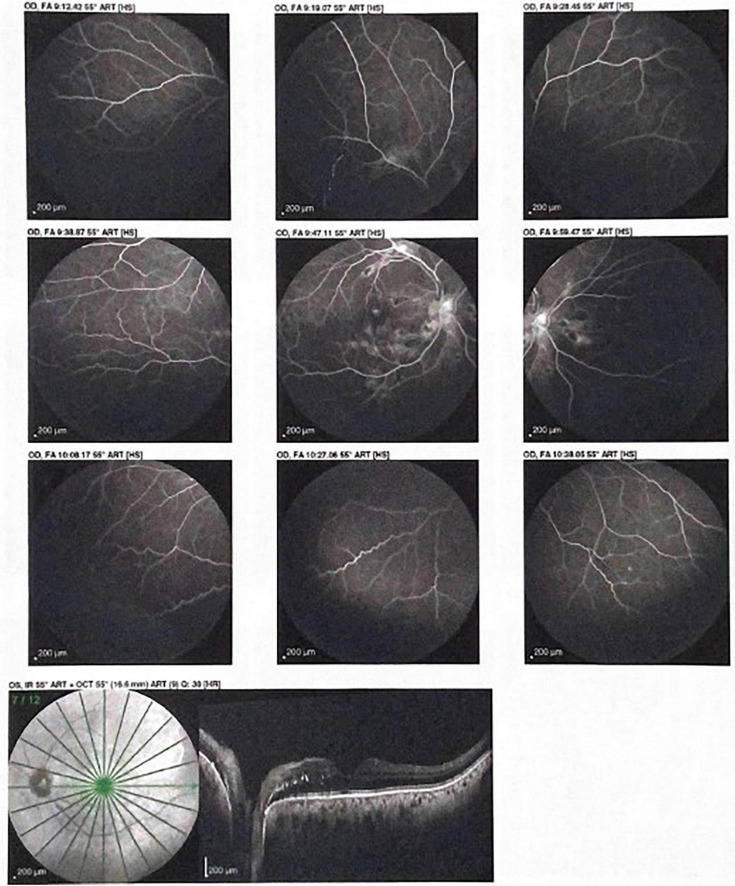
Optical coherence tomography (OCT) showing hard intra-retinal exudates with papilledema.

The funduscopic examination showed a large number of retinal flame hemorrhages located at peripapillar level. Retinal fluoroangiography and optical coherence tomography (OCT) ruled out ischemic areas, but additionally identified hard intra-retinal exudates with papilledema (Figure 3).

During the first 2 days of hospitalization, intravenous infusion of nitroglycerin and labetalol were maintained with a target systolic BP values between 150 and 160 mmHg. Thereafter intravenous therapy was replaced by transdermal nitroglycerin, and oral administration of amlodipine and carvedilol, with a progressive association with doxazosin.

A low dose of angiotensin-converting enzyme inhibitor (ACEi) (ramipril) was associated, despite the persistence of high creatinine values. Before discharge furosemide, atorvastatin, acetylsalicylic acid, a proton pump inhibitor, sodium bicarbonate and calcifediol were added to antihypertensive therapy.

The patient was discharged after a 40 days hospitalization [because of a concomitant paucisymptomatic Severe Acute Respiratory Syndrome–CoronaVirus2 (SARS-CoV-2) infection].

Genetic testing had been performed during the ICU hospitalization and nephrologist consultation in order to exclude atypical hemolytic uremic syndrome and Fabry disease (because of the suspicion of infiltrative hypertrophic cardiomyopathy).

After 120 days of follow-up, blood tests showed normal platelet count and hemoglobin level, but still increased creatinine (4.7 mg/dl).

Office BP values were high (180/115 mmHg) and the patient confirmed poor adherence to drug therapy. The importance of a regular treatment administration was reinforced to the patient and a 24 ABPM was performed, showing BP values still elevated but closer to target values, with dipping pattern.

**FIGURE 4:**
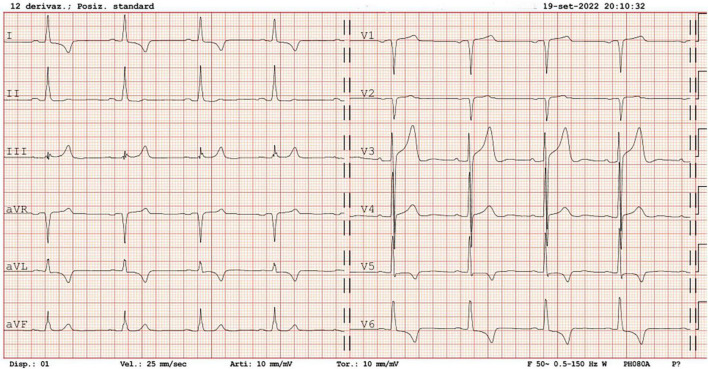
ECG at follow-up visit 4 months after discharge.

ECG showed reduction of the Sokolow–Lyon index (26 mm) and persistence of negative T waves (Figure 4).

**FIGURE 5:**
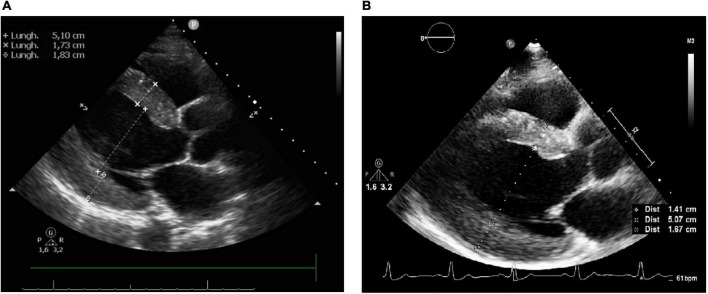
**(A)** Echocardiography after 2 months since the admission. **(B)** Echocardiography after 4 months since the admission.

An echocardiogram (performed 4 months after the admission) showed absence of wall motion abnormalities, normal LVEF (65%) and significant reduction of LVH (LV mass index 67.8 g/m^2.7^, IVS and PW thicknesses 14.1 and 16.7 mm, respectively), left atrial dilatation and slight increase of ascending aorta diameter (Figures 5A,B).

After 4 months from hospitalization a renal biopsy was performed showing findings suggestive of parenchymal renal damage and global glomerulosclerosis, possibly secondary to arterial hypertension. The renal biopsy excluded mesangial IgA deposits.

The patient is regularly seen in the outpatient clinic and will undergo a follow-up ECG and echocardiogram in the next months.

## Discussion

We report the case of a patient with malignant hypertension (MH). Several features suggest the diagnosis, including severe renal and cardiac damage, thrombotic microangiopathy and grade III-IV retinal fundus changes.

The patient clinical presentation was typical of a type 2 acute myocardial infarction (ST elevation in V1 and V2 associated with high troponin levels, wall motion changes and severe chest pain) due to moderate coronary angiography and increased oxygen demand and uptake (anemia and acute rise in BP). MH was initially underestimated and the diagnostic and therapeutic approach was first focused on the acute coronary syndrome hypothesis.

However, MH is not quite an obsolete diagnosis yet and the principles of early diagnosis, detailed systematic evaluation and effective treatment remain key factors for a longer event free survival.

After coronary angiography, specific tests were obtained to assess the cardiac, renal, and vascular damage, in addition to the exclusion of secondary forms of hypertension. ECG findings were typical of LVH secondary to hypertension, including both voltage and repolarization abnormalities such as the strain pattern. The hypothesis of Fabry’s disease proposed by the specialists who performed the first echocardiogram appears to be unlikely due to echocardiographic reduction of LV walls thickness at follow-up, obtained during an improvement in BP control.

More importantly we performed a retinal fundus examination that confirmed the presence of flame hemorrhages and a grade 3 Keith Wegener retinopathy, while papilledema was described at retinal fluoroangiography and optical coherence tomography. Unfortunately retinal fundus is still poorly prescribed in patients with acute BP elevation, despite new technical progresses that could widen its use (1, 2).

As suggested by guidelines (3, 4), the appropriate treatment involves early intravenous infusion of antihypertensive agents, although the decrease of BP should be particularly cautious and progressive. A decrease of mean blood pressure (MBP) of about 25% is suggested, to be reached in several hours.

In fact, the reduction in BP obtained in the ICU by nitroprussiate and nitroglycerin infusion induced a progression of myocardial damage, due to the reduction in coronary perfusion pressure, as described several years ago (5). In this patient the presence of LVH and of concomitant moderate atherosclerosis of large coronary arteries have further worsened myocardial damage.

A more permissive blood pressure target (160/95 mmHg) was achieved by reducing intravenous antihypertensive drugs, and a subsequent titration of oral treatment, allowing a more cautious and progressive reduction of blood pressure values.

There are no formal guidelines on the treatment of MH, due to the absence of randomized controlled clinical trials. The current consensus for treatment involves early intravenous infusion of antihypertensive agents, with a cautious and progressive decrease of BP. Labetalol, nicardipine, sodium nitroprusside and urapidil are the most often used and all appear to be safe and effective for the treatment of malignant hypertension (3). Whether intravenous therapy is always necessary is a matter of debate given recent reports that oral medication can also result in the controlled reduction of BP (6, 7). Oral therapy, in particular blockers of the renin–angiotensin system, may improve the management of MH. It offers a suitable treatment option in low-income countries where the condition remains relatively prevalent and in “uncomplicated” MH, oral treatments could be used to gradually lower BP to normal ranges over several days (6–10) (Table 1).

**TABLE 1:** Malignant hypertension therapy.

	Drugs	Onset	Half life	Dose	Contraindications/adverse effects
**IV drugs**					
First choice drugs	Labetalol	5–10 min	3–6 h	0.25–0.5 mg/kg i.v. bolus; 2–4 mg/min continuous infusion until goal BP is reached, thereafter 5–20 mg/h	**Contraindications:** history of 2nd or 3rd degree AV block, systolic heart failure, asthma, bradycardia, concomitant therapy with verapamil or diltiazem. **Protracted effect** in patients with liver dysfunction.
	Nicardipine	5–15 min	30–40 min	5–15 mg/h as continuous i.v. infusion, starting dose 5 mg/h, increase every 15–30 min with 2.5 mg until goal BP, thereafter decrease to 3 mg/h	**Adverse effects:** headache, tachycardia, hypotension, vomit. **Contraindications:** liver failure, patients with severe aortic stenosis. **Caution** with patients affected by acute heart failure.
	Nitroprusside	Immediate	1–2 min	0.3–10 mg/kg/min, increase by 0.5 mg/kg/min every 5 min until goal BP More than 2 mcg/kg/min can lead to thiocyanate toxicity (if necessary, use sodium thiosulfate)	**Adverse effects:** cyanide intoxication. **Relative contraindications:** liver and kidney failure. **Caution** with patients affected by intraparenchymal cerebral hemorrhage.
	Urapidil	3–5 min	4–6 h	12.5–25 mg i.v. bolus, 5–40 mg/h as continuous infusion	**Adverse effects:** nausea, dizziness, and headache. **Contraindications:** patients with severe aortic stenosis.
Second choice drugs	Nitroglycerine	1–5 min	3–5 min	Sublingual: 0.4 mg Continuous infusion: 5–200 mg/min, 5 mg/min increase every 5 min	**Adverse effects:** hypotension and reflex tachycardia. **Contraindications:** if cerebral or renal perfusion is compromised or if concomitant therapy with phosphodiesterase 5 inhibitors in the last 24–48 h.
	Esmolol	1–2 min	10–30 min	0.5–1 mg/kg i.v. bolus; 50–300 mg/kg/min as continuous i.v. infusion	**Contraindications:** history of 2nd or 3rd degree AV block, acute heart failure, asthma and bradycardia, concomitant therapy with verapamil or diltiazem. **Protracted effect** in patients with anemia.
**Oral drugs**					
	Amlodipine	4–8 h	24 h	5–10 mg Maximum dose: 10 mg/day	**Adverse effects:** peripheral oedema, headache, tachycardia, dizziness, nausea and vomiting, skin flushing.
	Doxazosin	Peak in 2 h	24 h	1–16 mg/day	**Adverse effects:** orthostatic hypotension, dizziness, retrograde ejaculation, priapism, oedema.
	Ramipril	1–2 h	24 h	2,5–10 mg 1–2 times/day	**Adverse effects:** hives, angioedema, hyperkalaemia, dysgeusia, vomiting, hacking cough, dizziness. **Recommended:** low dose in patients with kidney failure.

In this patient white matter lesions were observed in the frontotemporal lobes. Rubin et al. (8), following systematic evaluation of the brain by MRI, have observed that the white matter lesions were more prevalent in the posterior regions, with 51% of included patients affected. However, in 27% of the Bordeaux cohort other locations of white matter lesions were found.

This patient presented with renal damage and hypokalemia and in practice, these two findings with high BP should indicate malignant hypertension or TMA. Patients with malignant hypertension usually show hyperaldosteronism secondary to renin activation (8, 9), and the kidneys continue to excrete potassium.

A TMA was diagnosed and a genetic testing is ongoing to rule out the diagnosis of atypical hemolytic uremic syndrome.

The thrombotic microangiopathy can be caused by hemolytic uremic syndrome typical (HUS) and atypical (aHUS) or by thrombotic thrombocytopenic purpura (TTP or Moschcowitz disease) (10–12).

Thrombotic thrombocytopenic purpura was ruled out by the ADAMTS-13 assay (negative for mutations and the gene showed a physiological activity). The lack of clinical and anamnestic evidence for infection caused by Shiga toxin-producing *E. Coli* allowed to rule out the hemolytic uremic syndrome.

Other causes inducing TMA other than malignant arterial hypertension could be excluded (autoimmune diseases, exposure to trigger drugs, solid/hematopoietic organ transplantation, mesangial IgA deposits nephropathy, neoplasms, pregnancy/postpartum period). The atypical hemolytic uremic syndrome will be definitively excluded by the ongoing NGS genetic tests.

We followed the most recent recommendations for patients with MH (3, 4, 7–9). In the acute phase sodium nitroprusside, labetalol, nicardipine, and urapidil all appear to be safe and effective for the treatment of malignant hypertension; we did not use nicardipine because is not easily available in our country and we did not use diuretic therapy because of hypokalemia and pressure natriuresis. The choice of ACE inhibitor, despite the presence of severe increase in creatinine is suggested by some experts but should be started at a very low dose to prevent a sudden decrease in BP and then should be adjusted according to the patient’s response and the renal function (6). Patients with MH present with hypovolemia because of pressure natriuresis, and in some circumstances intravenous saline infusion can be used to correct precipitous BP reduction.

## Patient perspective

In patients with MH it is mandatory to prevent other clinical events, to carefully monitor blood pressure values and to avoid large and sudden decrease of BP in order to prevent further organ damage.

In this specific case, it was established to maintain a BP target above 140/85 mmHg to preserve renal and perhaps cardiac perfusion. Renal, cardiac and vascular organ damage should be checked by routine blood tests (including electrolytes and urinary proteinuria monitoring) in order to exclude possible complications of chronic kidney disease, ECG and echocardiogram and retinal fundus.

Adherence to antihypertensive therapy is crucial, as well as avoidance of non-steroidal anti-inflammatory drugs (NSAIDs) and of smoke.

In conclusion, the biggest challenge is to maintain the adherence to treatment and to the follow-up evaluation (13–16).

## Data availability statement

The original contributions presented in this study are included in the article/supplementary material, further inquiries can be directed to the corresponding author.

## Ethics statement

Ethical review and approval was not required for the study on human participants in accordance with the local legislation and institutional requirements. The patients/participants provided their written informed consent to participate in this study. Written informed consent was obtained from the individual for the publication of any potentially identifiable images or data included in this article.

## Author contributions

DM, FR, DS, CA, CD, MS, and MLM: clinical case management. FB, DM, EM, GM, FR, and NT: clinical data collection. MLM: manuscript revision and chief of the department. All authors contributed to the article and approved the submitted version.
